# Survey for Bat Lyssaviruses, Thailand

**DOI:** 10.3201/eid1102.040691

**Published:** 2005-02

**Authors:** Boonlert Lumlertdacha, Kalyanee Boongird, Sawai Wanghongsa, Supaporn Wacharapluesadee, Lawan Chanhome, Pkamatz Khawplod, Thiravat Hemachudha, Ivan Kuzmin, Charles E. Rupprecht

**Affiliations:** * Thai Red Cross Society, Bangkok, Thailand;; † Ministry of Agriculture, Bangkok, Thailand;; ‡ Chulalongkorn University, Bangkok, Thailand;; § Centers for Disease Control and Prevention, Atlanta, Georgia, USA

**Keywords:** Lyssavirus, rabies, RNA, bat, chiroptera, zoonosis, animals, fluorescent antibody technique, direct/veterinary, Thailand, research

## Abstract

Surveillance for lyssaviruses was conducted among bat populations in 8 provinces in Thailand. In 2002 and 2003, a total of 932 bats of 11 species were captured and released after serum collection. Lyssavirus infection was determined by conducting virus neutralization assays on bat serum samples. Of collected samples, 538 were either hemolysed or insufficient in volume, which left 394 suitable for analysis. These samples included the following: *Pteropus lylei* (n = 335), *Eonycteris spelaea* (n = 45), *Hipposideros armiger* (n = 13), and *Rousettus leschennaulti* (n = 1). No serum samples had evidence of neutralizing antibodies when tested against rabies virus. However, 16 samples had detectable neutralizing antibodies against Aravan virus, Khujand virus, Irkut virus, or Australian bat lyssavirus; all were specifically associated with fruit bats *P. lylei* (n = 15) and *E. spelaea* (n = 1). These results are consistent with the presence of naturally occurring viruses related to new putative lyssavirus genotypes.

Rabies is an acute encephalitis caused by a lyssavirus. On a global basis, bats have been associated with several different genotypes of lyssavirus (*1**–**5*). Two human infections with Australian bat lyssavirus (ABLV) have been reported, the clinical signs of which were consistent with classical rabies infection, namely a diffuse, nonsuppurative encephalitis (*3*). A serosurvey for agents similar to ABLV among bats in the Philippines detected a prevalence of 9.5% (22/231) (*6*). Six of 14 species (fruit- and insect-eating bats) were seropositive for reactivity against ABLV. These included *Taphozous melanopogan*(4/30), *Mineopterus schreibersi* (4/11), *Philetor brachypterus* (1/13), *Scotophilus kuhlii* (4/63), *Pteropus hypomelanus* (3/14), and *Rousettus amplexicaudatus* (6/50) (*6*). However, Asian bat lyssaviruses (*1**,**2**,**4*) were unavailable at that time to check for cross-reactivity.

Canine rabies is enzootic in Thailand. No bat-associated rabies or lyssavirus deaths in have been reported in humans or other animals (*7*).This lack of data for other agents, however, does not exclude their existence (*1*). Rabies statistics in humans and animals are underreported (*8*). Moreover, without a history of dog bite, rabies may be dismissed, or clinical manifestations of bat-related cases may be variable (*8*). In the context of bat lyssavirus as an emerging global infectious disease, baseline data are necessary to allow for future public health assessment of its impact. This active surveillance sought to determine whether bats in Thailand had evidence of lyssavirus infections.

## Methods

### Collection of Specimens

**Figure 1:**
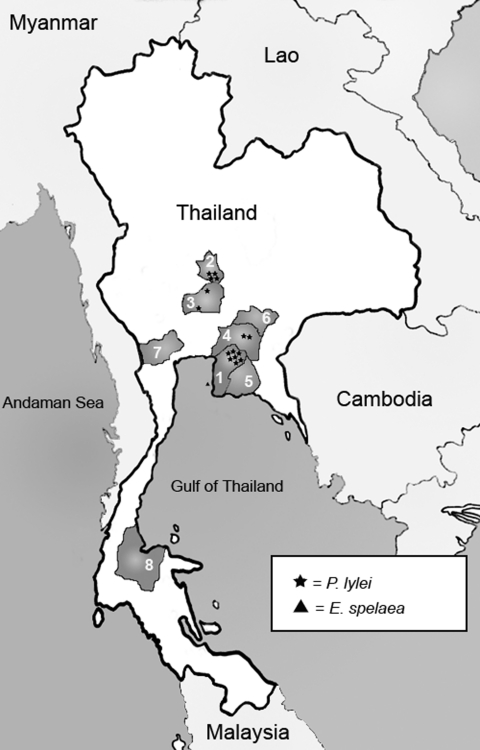
Map of Thailand showing bat collection sites from 8 provinces and locations of bats found seropositive. 1 = Chonburi, 2 = Singburi, 3 = Ayuttaya, 4 = Chachongsao, 5 = Rayong, 6 = Prachinburi, 7 = Ratchaburi, 8 = Suratthani.

**Figure 2:**
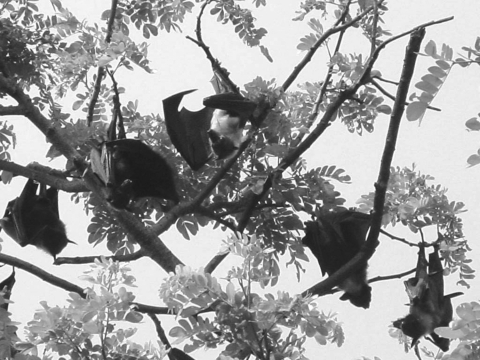
Thai Flying foxes (*Pteropus lylei*) at their roost.

From March 2002 through August 2003, bats were collected from 8 provinces throughout central, eastern, and southern Thailand (Figure 1). Sites were chosen on the basis of local reports of known bat colonies or after investigation by the Royal Department of Forestry, Ministry of Agriculture. Insectivorous bats in caves were captured during the day by using fine-mesh, long-handled butterfly nets. Larger fruit bats were captured with nets near sunset, as the bats flew for foraging activities, or before dawn when returning to their roosts (Figure 2). Thick leather gloves were worn when bats were handled and transferred into individual cotton pouches for transportation and processing.

Of the 932 bats collected, all were identified to 11 different species of both insectivorous and frugivorous bats (Table 1). Forty percent were female. All bats appeared healthy. At least 110 bat species (>20 million) are believed to be present in Thailand, according to estimates from a Royal Department of Forestry survey in 2003. Eighty five percent are insectivorous; the rest are frugivorous.

**Table 1:** Bat species captured in Thailand

Species	Province								
	Chonburi	Rayong	Ayuttaya	Chachoengsao	Singburi	Prachinburi	Ratchaburi	Suratthani	Total
*Hipposideros lavatus*	46							40	86
*H. armiger*							103		103
*Eonycteris spelaea*	28				36				64
*Rousettus leschennault*	1				5			5	11
*Pteropus lylei*	150		242	110	58	28			588
*P. hypomelanus*	16	3							19
*P. vampyrus*								23	23
*Emballonura monticola*								14	14
*Scotophillus heathi*			3						3
*Megaderma spasma*							13		13
*Cynopterus sphinx*							8		8
Total	241	3	245	110	99	28	124	82	932

Bats were anesthetized by administering a 0.2- to 0.5-mg intramuscular injection of ketamine hydrochloride. Animals were identified to sex and by species, based on gross morphology, as described (*9*). Animals were marked by hair or claw clipping. Blood, obtained from wing veins or by direct cardiac puncture, was transferred from the collecting syringe into 1.5-mL microtubes (Axygen Scientific, Union City, CA, USA) and stored in an icebox until centrifugation. Serum was frozen at –20°C during transportation and stored in a freezer at –70C. After recovery from sedation, bats were allowed to fly to their roosts. Sixteen of 932 died during the capture process. Inspection of the capture sites 1–2 months later included an assessment of whether the local ecology was disturbed. No additional bats died after the procedure, according to residents living near roosts.

### Serologic Testing for Neutralizing Antibodies

Serum specimens were obtained from blood samples after clotting. In general, 394 samples from 4 different species were of sufficient volume and quality (Table 2). The samples originated from Chonburi (n = 167), Ayuttaya (n = 105), Chachoengsao (n = 36), Singburi (n = 81), and Surattani (n = 5). For adequate volume during testing, they were diluted 1:5 in Eagle's minimum essential medium (Invitrogen, Carlsbad, CA, USA) supplemented with 2% fetal bovine serum (Invitrogen). Serum samples were heat-inactivated for 30 min at 56°C before testing.

**Table 2:** Bat sera screened and positive for neutralizing antibodies (positive/ screened)

Species	Site					
	Chonburi	Singburi	Ayuttaya	Chachoengsao	Suratthani	Total
*Hipposideros armiger*	1/8				0/5	1/13
*Eonycteris spelaea*	0/22	0/23				0/45
*Rousettus leschennault*	0/1					0/1
*Pteropus lylei*	8/136	4/58	2/105	1/36		15/335
Total	9/167	4/81	2/105	1/36	0/5	16/394

Initially, all 394 samples were screened in a modified rapid fluorescent focus inhibition test (*6*) against rabies virus (RABV, strain CVS-11) and ABLV (pteropid subtype; 40 50% tissue culture infective dose), with World Health Organization standard serum as a source for positive control antibody with 50% endpoint dilution of 1 IU = 1:20. Approximately 50 µL of diluted serum at 1:5, 1:10, and 1:20 dilutions was incubated with 50 µL of ABLV in 96-well microtiter plates for 90 min at 37°C in a CO_2_ incubator. Murine neuroblastoma cells (50 µL) were added to each serum-virus mixture, which was incubated for 20 h. Culture medium was removed after incubation, and the plates were fixed with 90% acetone, air-dried, and then stained with fluorescein isothiocyanate–conjugated anti-rabies monoclonal antibodies (Fujirebio Diagnostic, Inc, Malvern, PA, USA). Samples were considered positive if the number of fluorescent foci was reduced by 50% at the 1:5 dilution.

Those samples that demonstrated positive or suspicious activity were additionally tested against a broader panel of other lyssaviruses, including Aravan, Khujand, and Irkut virus isolates. Twofold serum dilutions, from 1:25 to 1:100, were tested, and virus doses varied from 32 to 100 infectious units. These reactions were performed by using drops of cell culture medium on 4-well (6-mm) Teflon-coated glass slides (Cell-line/Erie Scientific Co., Portsmouth, NH, USA), incubated in a moist chamber for 48 h.

### Direct Fluorescent Antibody (DFA) and Mouse Inoculation (MI) Testing of Brains

Brains from 16 dead bats (2 *P. lylei* and 14 *P. hypomelanus*) were collected in iceboxes at the capture sites for transportation and then were stored at –70°C until testing. Each brain was tested for lyssavirus antigen by DFA. Multiple impressions were prepared, and slides were fixed in acetone, allowed to dry at room temperature, and stained with commercial fluorescein isothiocyanate–conjugated anti-rabies monoclonal antibodies (Fujirebio Diagnostic, Inc). These brain impressions were examined with a fluorescent microscope.

For MI testing, pooled 20% brain suspensions from all 16 bats were prepared by mixing ≈0.5 g of each bat brain in 32 mL of normal saline solution. No antimicrobial preparations were added. The mixture was left to sediment at room temperature for 30 min, and the supernatant was used to inject into the brains of 1-month-old Swiss albino strain mice. Approximately 0.03 mL of each suspension was injected into each of 30 mouse brains. They were kept in 6 glass jars (5 in each) with a diameter of 15 cm and were observed for 60 days.

## Results

### Serologic Testing

All 394 serum samples were negative against RABV, but 16 (4%) were positive or suggestive of ABLV (Table 2). Further tests of these samples demonstrated neutralizing activity against Aravan, Khujand, or Irkut viruses or ABLV (Table 3). These 16 samples originated from 2 species, *P. lylei* (n = 15) and *Eonycteris spelaea* (n = 1), collected at Chonburi (n = 9), Singburi (n = 4), Ayuttaya (n = 2), and Chachoengsao (n =1) Provinces (Table 2).

**Table 3:** Neutralization of lyssaviruses by Thai bat sera*†

Serum ID	Antibody titers against different viruses				
	Aravan	Khujand	Irkut	ABLV	CVS-11
78B	<10	<10	**>200**	<10	<10
733	<10	<10	**>200**	<10	<10
688	**1:56**	**1:25**	<10	<10	<10
615	**1:13**	**1:25**	<10	<10	<10
120	<10	<10	**1:65**	<10	<10
0/69	<10	<10	**1:170**	<10	<10
96	<10	**1:21**	**1:33**	<10	<10
125	**1:20**	**1:56**	**1:13**	<10	<10
741	**1:12**	<10	**>200**	**1:13**	<10
731	<10	<10	**>200**	<10	<10
724	**1:35**	**1:56**	**1:29**	<10	<10
303	**1:35**	**1:50**	**1:50**	<10	<10
740	**1:35**	<10	**>200**	<10	<10
729	**1:40**	**>200**	**>200**	**1:20**	<10
461	**1:56**	<10	**1:16**	<10	<10
519	**1:35**	<10	**1:50**	<10	<10

*ABLV, Australian bat lyssavirus; CVS, challenge virus standard. †Boldface indicates statistical significance.

Chonburi is adjacent to Chachoengsao Province in the east, whereas Singburi and Ayuttaya are both located in the central part of the country (Figure 1). Approximately 5% of positive bat serum specimens were found in 2 eastern provinces (Chonburi, 9/158 and Chachoengsao, 1/36) versus 3% in 2 central provinces (Singburi, 4/81, and Ayuttaya, 2/105). Antibody-positive bats were dispersed throughout the collection period (March 2002 through August 2003). Most (15 of 16) positive samples came from *P. lylei*. One of 45 *E. spelaea* (versus 15 of 335 *P. lyle*i) tested positive.

### DFA and MI Testing

Sixteen bat brains tested by DFA had no detectable lyssavirus antigen. After intracerebral injection, 4 of 30 mice died, on days 11, 12, 14, and 21, respectively. None of these 4 brains tested positive with DFA for evidence of lyssavirus antigens.

## Discussion

This study presents evidence of neutralization of lyssaviruses other than RABV and ABLV by sera from Thai bats. These findings are consistent with the presence of naturally induced antibodies against >1 lyssavirus genotype in the Thai bat populations studied.

Lyssaviruses are classified into groups on the basis of their genetic, antigenic, and relative pathogenic attributes. At least 7 putative genotypes and 2 major phylogroups are recognized on the basis of their overall phylogenetic relatedness (*1*). Phylogroup I includes RABV (genotype 1), Duvenhage virus (DUVV) (genotype 4), European bat lyssavirus (EBLV) 1 (genotype 5), EBLV-2 (genotype 6), and ABLV (genotype 7). Phylogroup II includes Lagos bat virus (LBV) (genotype 2) and Mokola virus (MOKV) (genotype 3) (*10*). In this study, neutralization titers to new putative genotypes, namely, Irkut, Khujand, and Aravan viruses, and of much lesser degree to ABLV but not to RABV, were evident. Khujand virus is related to genotype 6, while Aravan virus is related to Khujand virus, with moderate similarity to genotypes 4, 5, and 6 (*2**,**4*). ABLV is more closely related to RABV (*3*). When a comparative phylogenetic analysis was performed, Irkut virus was recognized as a member of a cluster joining lyssavirus genotypes 4 and 5 (76% bootstrap support) (*1*).

This preliminary study demonstrates that which virus is used for a serologic test is critical. All Thai samples were negative to RABV and most to ABLV, findings which help explain why lyssavirus infection has not previously been reported in Thai bats. A relatively low prevalence of lyssavirus infection in Thai bats in the current study (4% as compared to 9.5% in the Philippines survey [6]) may be explained by the fact that as many as 43 samples had a 1:5 (some of them, both 1:5 and 1:10) dilution considered unreadable because of the effect of hemolysis. Moreover, another 13 samples with equivocal result were seropositive for ABLV after subsequent testing. Further testing of these additional 13 samples against Irkut, Khujand, and Aravan viruses was not possible because of insufficient volume. Therefore, the actual positive number might be 29 (7.3%) of 396. Nevertheless, without a Thai lyssavirus isolate, concluding to which virus these bats have been exposed is difficult. These data also suggest that several lyssaviruses are in circulation throughout Thailand as well as other Asian countries, such as in the Philippines, Central Asia, and portions of Russia (*1**,**2**,**4**,**6*).

Further studies throughout the year should be expanded to other species of bats, as well as a focus upon bats such as *P. lylei* and in locations with the highest prevalence of neutralizing antibodies. Whether *P. lylei* is the single most important species is not known. Surveillance among sick and dying bats and collection of their brains would assist in identifying infecting viruses.

Public health authorities need to be aware of the potential for bats to transmit lyssaviruses, and to increase surveillance and public education. Attention should focus on the protective efficacy of commercially available vaccines and immune globulins against these novel nonrabies lyssaviruses after exposure, before fatal human infection occurs.
